# [18F]-Fluorodeoxyglucose-Positron Emission Tomography in Rats with Prolonged Cocaine Self-Administration Suggests Potential Brain Biomarkers for Addictive Behavior

**DOI:** 10.3389/fpsyt.2017.00218

**Published:** 2017-11-01

**Authors:** Nazzareno Cannella, Alejandro Cosa-Linan, Mareike Roscher, Tatiane T. Takahashi, Nils Vogler, Björn Wängler, Rainer Spanagel

**Affiliations:** ^1^Medical Faculty Mannheim, Institute of Psychopharmacology, Central Institute of Mental Health, Heidelberg University, Mannheim, Germany; ^2^Medical Faculty Mannheim, Department of Clinical Radiology and Nuclear Medicine, Heidelberg University, Mannheim, Germany

**Keywords:** [18F]-fluorodeoxyglucose-positron emission tomography, cocaine, addiction, neural activity, biomarker, glucose metabolism, relapse, psychostimulants

## Abstract

The DSM5-based dimensional diagnostic approach defines substance use disorders on a continuum from recreational drug use to habitual and ultimately addicted behavior. Biomarkers that are indicative of recreational drug use and addicted behavior are lacking. We performed a translational [18F]-fluorodeoxyglucose-positron emission tomography (FDG-PET) study in the multi-dimensional 0/3crit model of cocaine addiction. Addict-like (3crit) and non-addict-like (0crit) rats, which shared identical life conditions and levels of cocaine self-administration, were acquired for FDG-PET under baseline conditions and following cocaine and yohimbine challenges. Compared to cocaine-naïve control rats, 0crit animals showed higher glucose uptake in the caudate putamen (CPu) and medial prefrontal cortex (mPFC) respect to naïve controls. 3crit animals did not show this adaptive higher glucose utilization, but had lower uptake in several cortical areas. Both cocaine and yohimbine challenges affected glucose uptake in control rats in several brain sites, but not in 0crit and 3crit rats, indicating that impaired glucose mobilization in response to these challenges is not specifically associated with addictive behavior. Compared to 0crit, 3crit rats showed higher reinstatement responses, which were negatively associated with glucose uptake in the ventral tegmental area. Data indicate that cocaine non-addict- and addict-like phenotypes are associated with several potential biomarkers. Specifically, we propose that increased glucose uptake in the CPu and mPFC is a function of controlled drug use, whereas a loss of striatal and prefrontal metabolic activity and reduced uptake in cortical areas are indicative of addictive behavior.

## Introduction

Cocaine addiction is a psychiatric disorder characterized by the maintenance of drug use despite detrimental consequences. The loss of control over drugs of abuse is associated with altered brain functions revealed by several neuroimaging techniques. Together with the glucose-homologous radiotracer [^18^F]-fluorodeoxyglucose (FDG), positron emission tomography (PET) is a valuable tool with which to identify impaired brain glucose metabolism associated with psychiatric conditions such as addiction (1). In the past, FDG studies have demonstrated abnormal brain glucose metabolism associated with cocaine addiction and withdrawal. Acute withdrawal in cocaine-abusers is associated with a glucose metabolic rate higher than drug-naïve controls or cocaine abusers tested in late withdrawal (2); in addition, severity of cocaine use is negatively correlated with glucose metabolic rate (3) and cocaine abstinence is associated with decreased cortical glucose metabolism (4). FDG-PET was also used to investigate brain glucose metabolic rate associated with relapse factors such as drug-related cues (5–9) and the acute effect of a cocaine challenge (10, 11). However, in contrast to clinical studies on drug-related cues, ethical concerns hamper human studies on the effect of cocaine challenges to drug-naïve subjects (10). This limits the understanding of the physiological and abnormal responses to drugs, as healthy drug users are usually chosen as controls. Another drawback of FDG-PET studies conducted to date is the lack of non-addict drug user controls that allow the disentangling of adaptations associated with loss of control from controlled cocaine use. Such a comparison is critical for the identification of potential biomarkers.

Preclinical cross-sectional studies help overcome the limitations of clinical research comparing controlled experimental conditions impossible to obtain in humans [(12); see also comparison of short and long cocaine access in Ref. (13)]. Therefore, we used the multi-dimensional 0/3 criteria (0/3crit) rat model of cocaine addiction (14) to study differences in brain glucose metabolic rate between addict-like (3crit), non-addict-like (0crit) and cocaine-naïve age-matched control rats. Similar to prevalence rates of cocaine addicted individuals (15), 15–20% of a rat cohort loses control over cocaine-seeking and intake after prolonged training (3crit) in the 0/3crit model of addiction, whereas the majority of animals maintain control over cocaine (0crit). Another valid preclinical model of loss of control over cocaine is the long-access vs short-access model (16). This model has been recently combined with FDG-PET imaging to study brain glucose uptake in rats undergoing escalation of cocaine intake (13). Here we focused on individual differences between subjects showing addict-like and non-addict-like behavior that are independent from differences in cocaine intake. For this purpose, we used FDG-PET to compare brain glucose uptake in 0crit vs 3crit vs cocaine-naïve age-matched controls under baseline conditions and following pharmacological challenges with cocaine. In addition, being cocaine a non-selective monoamine transporter blocker (17), other monoamines such as norepinephrine could contribute to its effect on glucose metabolism. Therefore, we tested also a pharmacological challenge with yohimbine, because yohimbine, as a α2 adrenergic receptor antagonist, indirectly elevates norepinephrine levels and it is a pharmacological stressor able to reinstate drug-seeking (18–21).

## Materials and Methods

Experimental procedures were in accordance with the NIH ethical guidelines for the care and use of laboratory animals and conform to the EU Directive 2010/63/EU for animal experiments, and were approved by the local animal care committee (Regierungspräsidium Karlsruhe, Germany).

### Subjects

Fifty-six male Sprague-Dawley rats (Charles River, Germany), beginning self-administration training at 11 weeks, were individually housed on a reversed light cycle in three UniProtect air-flow cabinets (Bioscape, Germany), located in a temperature (22 ± 1°C) and humidity (60 ± 5%) controlled room. Eight rats were randomly selected to constitute the cocaine naïve age-matched control group (AC), while 48 entered cocaine self-administration (CSA) training. During training and tests, subjects received 20 g/day of standard chow food, water was provided *ad libitum*.

### Surgeries

Rats were implanted under isoflurane anesthesia with a catheter composed of a Micro-Renathane^®^ tube (internal diameter: 0.58 mm; external diameter: 0.94 mm) and a back-mount (Plastic-One). The proximal end reached the right atrium through the right jugular vein, whereas the back-mount passed under the skin and protruded from the mid-scapular region. Rats were given 9–14 days recovery before CSA sessions began. Catheter were flushed before and after each self-administration session during cocaine training and every second day during PET acquisition with a heparinized solution (100 IU/ml) containing 1 mg/ml of enrofloxacin (Baytril^®^).

### Drugs and Chemicals

Cocaine-HCl, yohimbine-HCl (Sigma-Aldrich, Germany) and [Fluorine-18]-fluorodeoxyglucose (FDG) (ZAG Zyklotron AG, Karlsruhe, Germany) were dissolved in sterile saline and administered intravenously (i.v.) *via* the implanted catheter.

### Self-Administration Apparatus

Behavioral training and tests were run in 24 self-administration chambers (40 cm long × 30 cm width × 52 cm high) located in sound-attenuating cubicles equipped with exhaust fans to assure air renewal. Two poke holes were on opposite walls of the chambers, 5 cm above the grid floor. When rats poked their snout in the holes, breaking an infrared beam, their instrumental responding was recorded. One hole was associated with cocaine delivery and designated as the active hole, while the other was designated as the inactive hole and served as control. A white house light located at the top allowed the illumination of the whole chamber, a white cue light was located 9.5 cm above the active hole, a green cue light was 10 cm to the right of the white one, and a blue cue light was on the left side of the opposite wall 33 cm above the grid floor. Experiments were controlled and data collected with Windows-compatible SK_AA software (Imetronic, France).

### 0/3crit Model of Addiction

As we previously described (22, 23), all procedures were adapted from previous publications using the 0/3crit model (24–29).

#### Basal Training Protocol

Rats performed CSA training 5 day/week. Sessions were composed of three drug periods (40 min each) separated by two no-drug periods (15 min each). “Drug” periods were signaled by the blue cue light, whereas “no-Drug” periods were signaled by the illumination of the entire chamber by the house light. During “Drug” periods, following the required amount of nose-pokes in the active hole, the white cue light was illuminated, and after 1 s the infusion pump was activated (40 μl/infusion over 2 s, containing 0.8 mg/kg of cocaine). The white cue light remained on for 4 s in total. Cocaine infusion was followed by a 40 s time out period. During the first 3 days a fixed ratio (FR) 3 schedule of reinforcement was applied. Then, the schedule was increased to FR5 for the remainder of training. In each CSA session, except those during which motivation for cocaine-taking, resistance to punishment, and *ad libitum* cocaine intake were tested, a maximum of 35 infusions was allowed. Nose-poking in the inactive hole was recorded but had no programmed consequences. During the “no-Drug” periods, nose-poking in both holes was recorded but had no programmed consequences. When rats had performed a minimum of 48 CSA sessions, three criteria for addiction-like behavior were tested: persistence of drug-seeking, motivation for cocaine-taking, and resistance to punishment.

#### Persistence of Drug-Seeking

This addiction criterion was evaluated daily during basal training by summing up the nose-pokes in the active hole during the two “no-Drug” periods, when cocaine was not available and accordingly signaled. The average across the last three basal training sessions before motivation test (see below) was considered for analysis.

#### Motivation for Cocaine-Taking

This addiction criterion was evaluated after 48 days of training using a progressive ratio (PR) schedule of reinforcement. During the PR session, the environmental setup was identical to a standard “Drug” period except that the ratio of response was increased after each infusion according to the following progression: 10, 20, 30, 45, 65, 85, 115, 145, 185, 225, 275, 325, 385, 445, 515, 585, 665, 745, 835, 925, 1,025, 1,125, 1,235, 1,345, 1,465, 1,585, 1,715, 1,845, 1,985, 2,125, 2,275, 2,425, 2,585, 2,745, 2,915, 3,085, 3,265, 3,445, 3,635, 3,825, 4,025, 4,225. The last ratio completed is referred to as the break point (BP) and used to score the criterion. The session ceased after 5 h or whether 1 h passed from the last infusion earned (i.e., after an infusion the rats had 1 h to produce the number of active pokes required to earn the next).

#### Resistance to Punishment

This criterion was evaluated after 52 days of training by pairing cocaine-seeking and taking with electric foot shocks, as described below. The session lasted 40 min and was a modified version of the standard “Drug” period. As usual, rats were required to achieve an FR5 ratio of responses to receive the cocaine injection. However, in this case, FR1 led to the illumination of the green cue light, signaling the presence of foot-shock. When FR4 was completed, rats received an electric foot-shock (0.2 mA for 1 s). When FR5 was completed rats received both a foot-shock (0.2 mA 1 s) and a cocaine infusion (0.8 mg/kg). Rats had 1 min to reach FR4 and then 1 min to complete FR5; if these requirements were not met, the green cue light was extinguished and the sequence reinitiated. The blue and white cue light schedules functioned as in the standard “Drug” period. This criterion was expressed as percentage of cocaine infusions earned respect to the baseline training.

#### Characterization of Addiction-Like Behavior

A subject was considered positive for one criterion if its score was above the 60th percentile of the population distribution, and negative if its score was below the 60th percentile (14, 30). Thus, depending on the number of positive criteria met, a subject was assigned to one of the following groups: 0crit, 1crit, 2crit, and 3crit. Rats negative for all the criteria (0crit) were characterized as non-addict-like, whereas rats positive for all the criteria (3crit) were characterized as addict-like.

### Cocaine-Induced Reinstatement

After PET acquisitions, rats performed a minimum of seven baseline CSA sessions before entering the two-day reinstatement test adapted from Ref. (24). Each session lasted for 210 min. On session 1, following a 90-min period of extinction, rats received four intravenous boluses (20, 40, 80, and 160 µL in ascending order) of vehicle at the ratio of one infusion every 30 min. On session 2, the same schedule was applied except that the vehicle solution was replaced by a cocaine solution (identical to CSA session), leading to cocaine priming doses of.4, 0.8, 1.8, and 3.2 mg/kg. Thus, the reinstatement phase corresponded to the 2 h following the delivery of the first cocaine bolus. Rats’ nose-pokes in both active and inactive holes were recorded but had no scheduled consequences.

### Yohimbine-Induced Reinstatement

After cocaine-induced reinstatement, rats repeated standard CSA baseline for 1 week before a yohimbine-induced reinstatement test. This test was adapted from the 2-day protocol used for cocaine-induced reinstatement. Each session lasted 180 min. During session 1, following a 90-min period of extinction, rats received an intravenous bolus (80 µL) of vehicle, after which the session continued for the next 90 min. During session 2, the same schedule was applied except that the vehicle solution was replaced by a yohimbine solution (0.5 mg/kg) (21). The reinstatement phase corresponded to the 90 min following the delivery of the yohimbine bolus. Rats’ nose-poking in both active and inactive holes were recorded but had no scheduled consequences.

### Selection of Rats for PET Acquisition

AC, 0crit and 3crit, were recruited for PET acquisitions. The 0/3crit model of addiction yield about 15–20% of 3crit and about 35–40% of 0crit out of the entire rat cohort; therefore, all 3crit animals available were recruited; only eight out of 15 0crit were selected to match group sizes. To select the eight 0crit for PET acquisition, we used the addiction score, a dimensional index of cocaine use severity linearly related to the number of criteria met (22, 26). Given the skewed distribution of the three addiction-like criteria, data were log-transformed prior to the computation of addiction score, defined as the algebraic sum of the z-scores of each of the three addiction-like criteria.

### Experimental Timeline

After rats were characterized for their addiction-like behavior, 0crit (Non-Addict-Like), 3crit (Addict-Like), and AC rats were selected for PET acquisitions. Baseline training was maintained until 0crit (*N* = 8), 3crit (*N* = 8), and AC (*N* = 8) were subjected to PET acquisitions. Each subject received three FDG-PET scans. The first two scans were used to test baseline and cocaine-induced activity in counter-balanced order; the third scan was used for yohimbine-induced activity. The number of rats scanned each day was balanced across groups. Between acquisition sessions rats remained in their home cage. After the three PET acquisitions, rats performed a new baseline training before cocaine- and yohimbine-induced reinstatement tests. As it is known that many different factors like anesthesia, circadian rhythm, body temperature, stress, blood glucose level, and route of FDG administration can alter the uptake of FDG (31–34), a standardized protocol was used to keep the acquired data comparable and meaningful. Measurements were always held in the morning, in rat’s waking phase. Deleye and colleagues (34) showed that a fasting period of 12 h ensures a considerable pre-scan glucose level so that no glucose level correction needs to be performed. The animals in our study were fed restrictively with 20 g/day of standard chow pellets every morning so that no definite fasting period could be determined, although by experience we can assume that the fasting period was >12 h. Therefore, blood glucose levels were measured pre- and post-scan and the data were corrected for blood glucose levels. Also in the course of long-term studies with repeated measurements of the same animal, intra- and inter-individual variation might lead to a loss of statistical power. Hence, the inter-scan period was set for at least 48 h in order to reduce the influence of previously observed significant variations in FDG brain uptake values due to changes in blood glucose level (34).

### PET Acquisition and Processing

[18F]-Fluorodeoxyglucose was used to measure the effects of acute cocaine or yohimbine administration in comparison to baseline activity in the three groups. Depending on scan session, either saline, cocaine (0.8 mg/kg), or yohimbine (0.5 mg/kg) solution was freshly prepared and co-injected with FDG. In order to correct the data for intra-individual variation in blood glucose levels during the course of the subsequent scans, blood glucose levels were determined in duplicate from a drop of blood from the tail vein before injecting the FDG and after the PET/CT scan using a blood glucose meter with strips (GlucoCheck XL; Aktivimed GmbH, Rheine, Germany; 0.5 µL blood). FDG (mean 28.25 ± 2.95 MBq; range between ~23 and 33 MBq) was administered *via* the injection port after flushing the line with heparinized solution prior to and following radioactivity administration. For optimal tracer distribution, we kept rats conscious for the 30-min uptake phase in their home cages, where they remained undisturbed. Afterward, anesthesia was initiated and maintained for the duration of the scan using isoflurane (2–3.5% delivered at 2.5 L/min). A static PET scan starting 40 min post injection was performed over 30 min, and a subsequent CT image was acquired using the tri-modal Bruker Albira small-animal PET/SPECT/CT (Bruker Biospin GmbH). PET reconstruction was performed using maximum likelihood expectation maximization algorithm with a matrix size of 20 × 20 and a pixel size of 0.5 mm (12 iterations) with the Albira Suite Reconstructor (Bruker Biospin MRI GmbH, Ettlingen, Germany), with data output in kBq/cc. The acquired data were corrected for scatter, attenuation, and dead-time. Registered PET images were normalized and transformed to the space of a standard FDG-PET template (32) using brain normalization in PMOD v3.6 (PMOD Technologies, Zurich, Switzerland). The MRI brain whole PET VOI template co-registered to the *a priori* FDG-PET template available in PMOD was used for analysis of the PET data.

### Image Analysis

Reconstructed PET images reflecting cerebral metabolic rate of glucose (CMRGlu) were spatially normalized to the FDG-PET template for rats by affine transformation and standardized uptake values (SUV) were calculated. To adjust inter-individual differences in global uptake due to physical and biological sources of variability, SUV maps values were scaled by adjusting the mean based on the average of SUV within the entire brain mask that excludes regions outside the brain (SUVR). Note that SUVR equals the globally normalized CMRGlu by canceling the injected radiotracer and the body weight factors in the calculation of SUV. Fifty-eight predefined regions of interest (ROIs) encompassing the entire brain were defined and the regional mean SUVR was calculated.

### Statistical Analysis

Group scores for each addiction-like criterion (motivation for cocaine-taking, persistence of cocaine-seeking, resistance to punishment) and total number of cocaine injections self-administered throughout the training by 0crit and 3crit rats were analyzed by Student’s *t*-test. Reinstatement tests were analyzed by a three-way ANOVA with group as a between factor, and nose-pokes (active/inactive) and 30 min time bins (last 30 min of extinction and reinstatement phase, note that for cocaine priming each time bin correspond to a priming dose) as repeated measures; ANOVA was followed by Newman–Keuls *post hoc* analysis when appropriate.

The impact of cocaine and yohimbine on regional brain glucose uptake was analyzed with a two-way mixed ANOVA. The model included the injection (two levels, saline and cocaine/yohimbine injection) as a within-subject factor and group as between-subject factor (3 levels, 0crit, 3crit, and age control). For each test, statistical significance was set at *p* < 0.05, followed by *post hoc* pairwise comparison analysis.

To examine whether the severity of the addiction (three addiction sub-dimensions and the addiction score) influences the glucose uptake under pharmacological manipulations, and whether glucose uptake correlates with the reinstatement ratio, a Pearson’s correlation was computed for each of the cocaine-exposed groups (0crit and 3crit animals). The correlation was considered significant if the *p* value did not exceed the Bonferroni correction across behavioral measures (*p* < 0.05 uncorrected; *p* < 0.0083 corrected).

## Results

### Characterization of Addiction-Like Behavior

**Figure 1:**
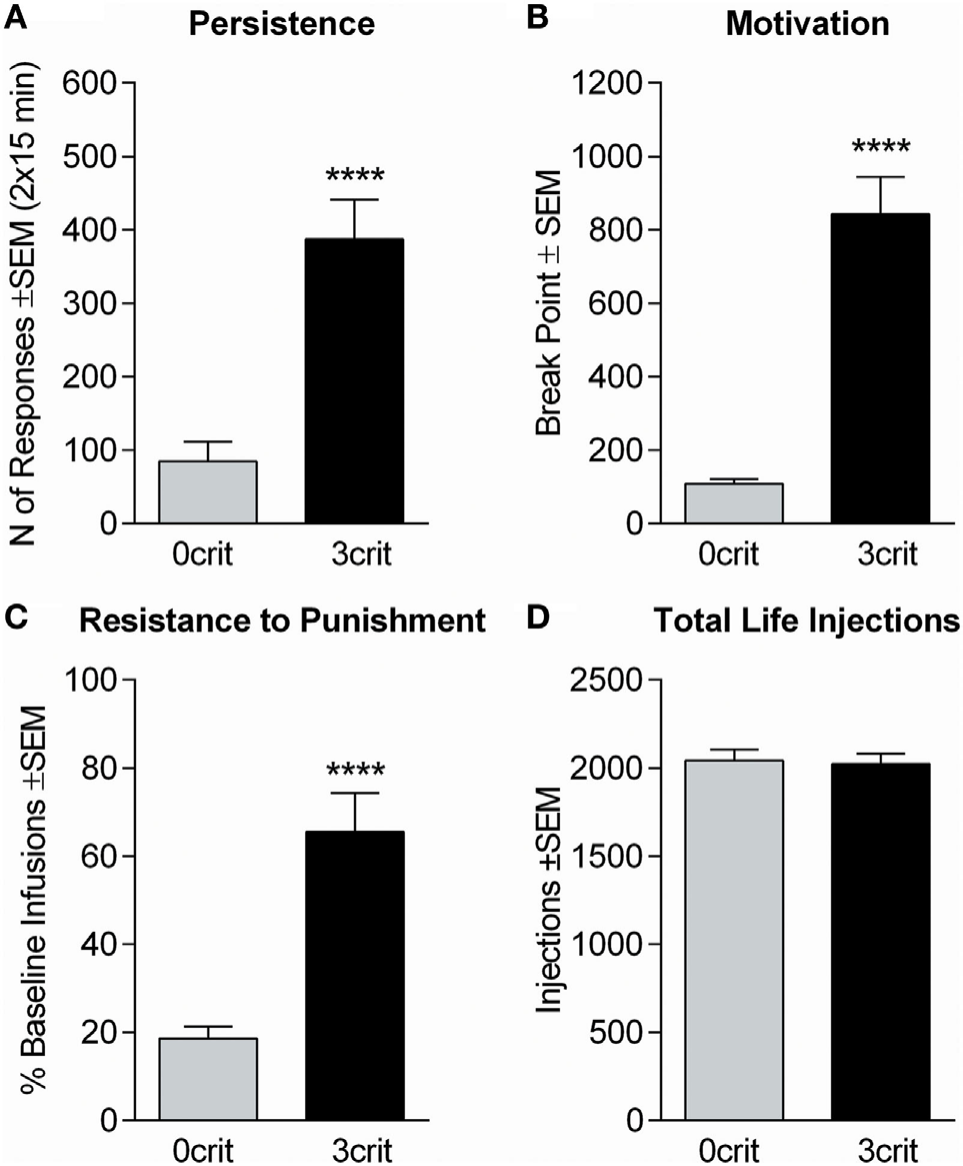
Behavioral characterization of 0crit and 3crit rats selected for positron emission tomography (PET) acquisition. 3crit (*n* = 8) rats showed higher response than 0crit (*n* = 8) in each addiction-like criterion. **(A)** Persistence of cocaine-seeking expressed by the sum of active nose-pokes during the no-drug periods averaged across sessions 45–47. **(B)** Motivation for cocaine intake expressed as the break point during a progressive ratio session performed on session 48. **(C)** Resistance to punishment expressed as percent of baseline cocaine infusions when cocaine-seeking and -taking are paired with a foot-shock punishment; the test was performed on session 52, the number of infusions earned during the first drug-period averaged across sessions 49–51 were considered as baseline. **(D)** Total number of cocaine injections self-administered by 0crit and 3crit before PET acquisition, the two groups did not differ in number of cocaine injection self-administered. Data are shown as mean ± SEM; ****p < 0.0001 respect to 0crit.

Of 48 rats entering CSA training, 5 were excluded due to catheter failure. Based on the number of positive and negative criteria met, the 43 remaining subjects yielded 15 0crit, 11 1crit, 9 2crit, and 8 3crit. 0crit were those subjects scoring below the 60th percentile in each criterion and defined as non-addict-like, whereas 3crit were those subjects scoring above the 60th percentile in each criterion and defined as addict-like. All 3crit rats were recruited for PET acquisition. Of the 15 0crit, eight were selected for PET acquisition. When analyzing addiction-like criteria, we found, as expected, that 3crit scored higher than 0crit in each criterion (behavioral sub-dimension): persistence of drug-seeking [*t*(14) = −5.04, *p* = 0.00018; Figure 1A], motivation for cocaine [*t*(14) = −7.11, *p* = 0.000005; Figure 1B] and resistance to punishment [*t*(14) = −5.07, *p* < 0.00017; Figure 1C]. However, 0crit and 3crit did not differ in total number of cocaine injections self-administered throughout training [*t*(14) = −0.2, *p* = 0.84; Figure 1D]. In conclusion, 0crit and 3crit rats shared identical life conditions and levels of cocaine exposure and can therefore be used for direct comparison of controlled vs addict-like drug consumption.

### Group and Drug-Induced Differences in SUV Reveal Potential Biomarkers

**Figure 2:**
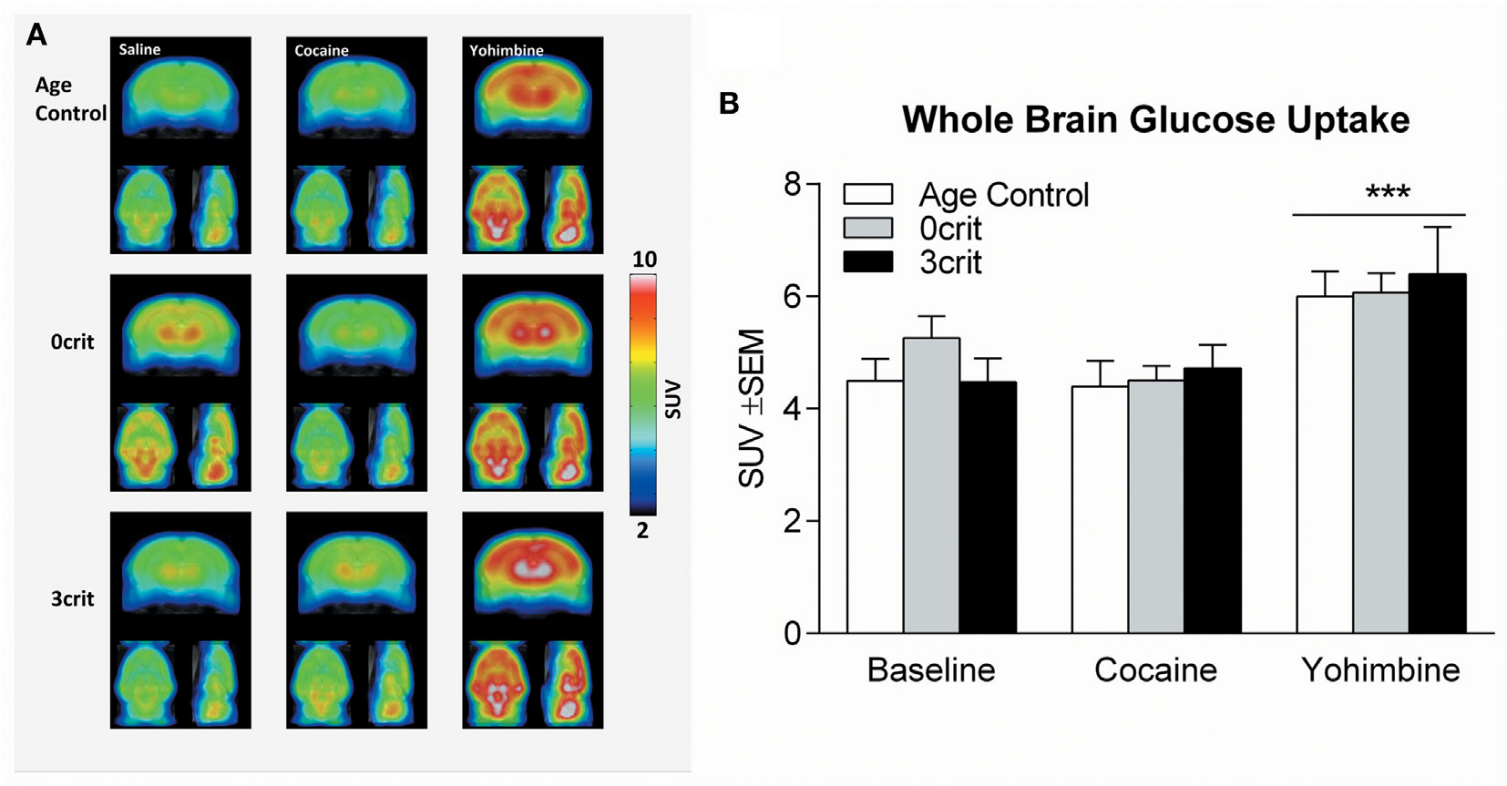
Whole brain glucose uptake in Age Control, 0crit and 3crit rats. **(A)** Average reconstructions of microPET scans showing [18F]-fluorodeoxyglucose (FDG) uptake in Age Control (upper row), 0crit (middle row), and 3crit (lower row) under baseline (left column) condition and cocaine (middle column) or yohimbine (right column) treatment. **(B)** Standardized uptake value (SUV) of the whole brain after injection of FDG in combination with saline or cocaine or yohimbine in Age Control, 0crit, and 3crit rats. ***p < 0.001 vs baseline.

One 3crit rat died under anesthesia. Therefore, PET acquisitions were performed in seven of the eight 3crit. In the analysis of the average whole brain uptake under baseline and cocaine challenge conditions, ANOVA found no effect of group (*F* = 0.513, *p* = 0.607), drug (*F* = 0.514, *p* = 0.482) or group by drug interaction (*F* = 1.2, *p* = 0.324), (Figures 2A,B). In the analysis of the average whole brain uptake under baseline and yohimbine challenge conditions, ANOVA found no effect of group (*F* = 0.257, *p* = 0.776) but a significant effect of drug (*F* = 17.1, *p* = 0.001). However, the drug by group interaction was not significant (*F* = 0.970, *p* = 0.398), suggesting that the effect of yohimbine was independent of group (Figure 2B).

**Figure 3:**
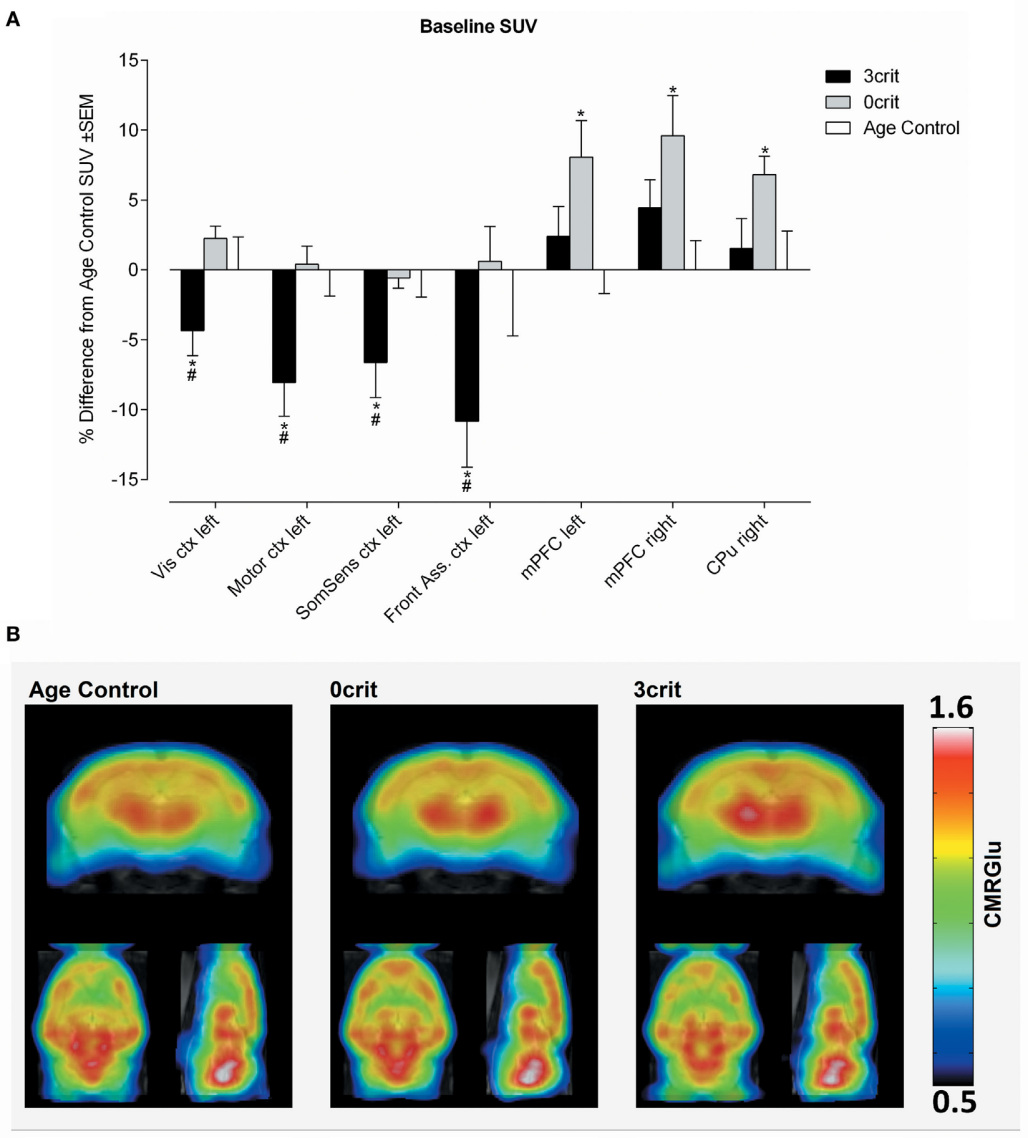
Regional quantification of metabolic activity under baseline condition. **(A)** Baseline SUV expressed as relative difference from Age Control measured after i.v. injection of a saline bolus. 3crit rats show a lower SUV in several cortices, conversely in 0crit have higher SUV in the mPFM and CPu respect to naïve rats. **(B)** Metabolic maps representing baseline cerebral metabolic rate of glucose (CMRGlu) in Age Control, 0crit, and 3crit rats. *p < 0.05 vs Age Control, #*p* < 0.05 vs 0crit. CPu, caudate putamen; mPFC, medial prefrontal cortex; Front Ass ctx, frontal association cortex; SomSens ctx, somatosensory cortex; Motor ctx, motor cortex; Vis ctx, visual cortex.

**Figure 4:**
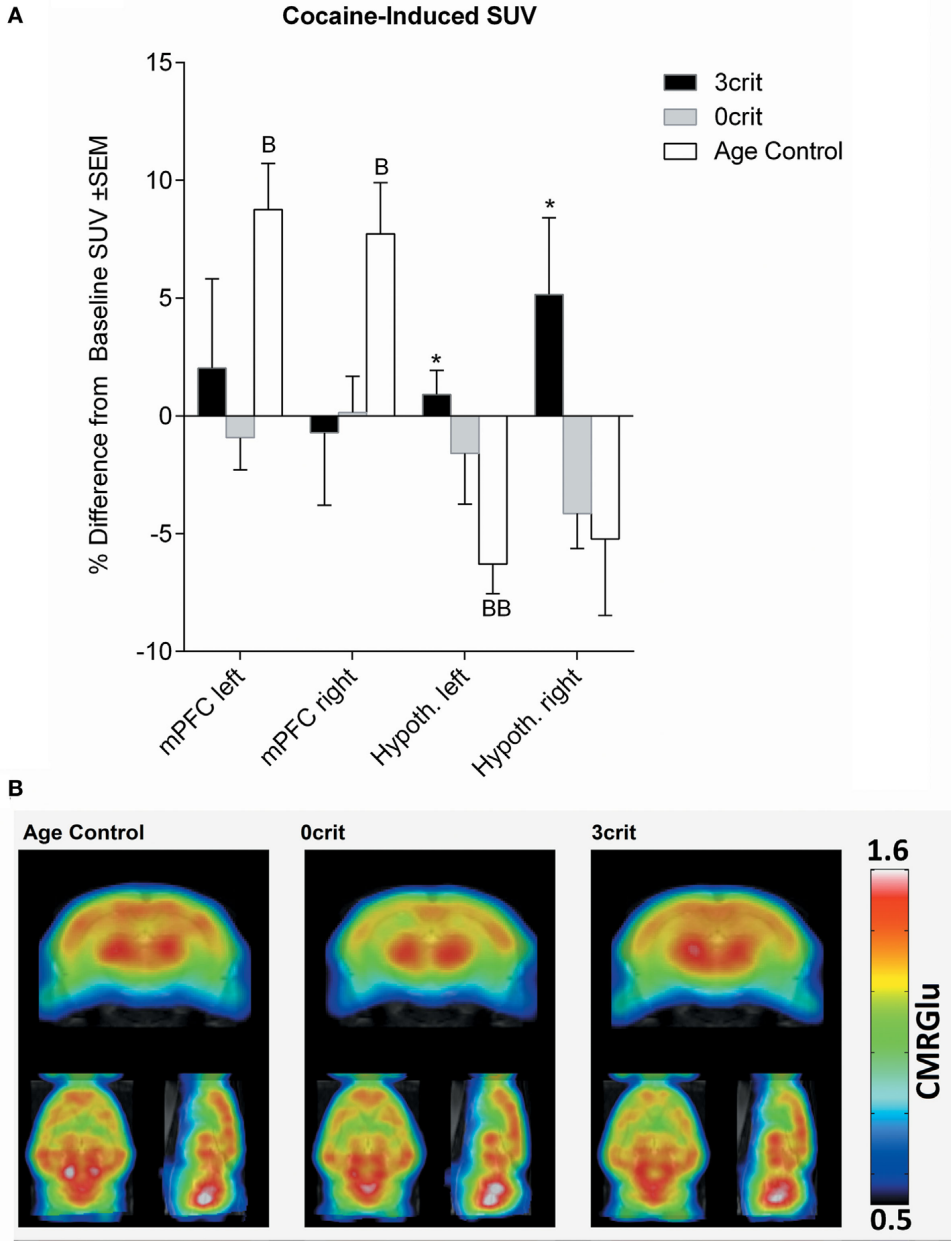
Regional quantification of metabolic activity after infusion of a cocaine bolus (0.8 mg/kg i.v.). **(A)** Cocaine-induced SUV expressed as relative difference from baseline (i.e., SUV measured after saline injection). Cocaine increased SUV in the mPFM and decreased it in the hypothalamus of cocaine naïve rats. **(B)** Metabolic maps representing cocaine-induced cerebral metabolic rate of glucose (CMRGlu) in Age Control, 0crit, and 3crit rats. *p < 0.05 vs Age Control, B *p* < 0.05 and BB *p* < 0.01 vs Baseline SUV. mPFC, medial prefrontal cortex; Hypoth, hypothalamus.

**Figure 5:**
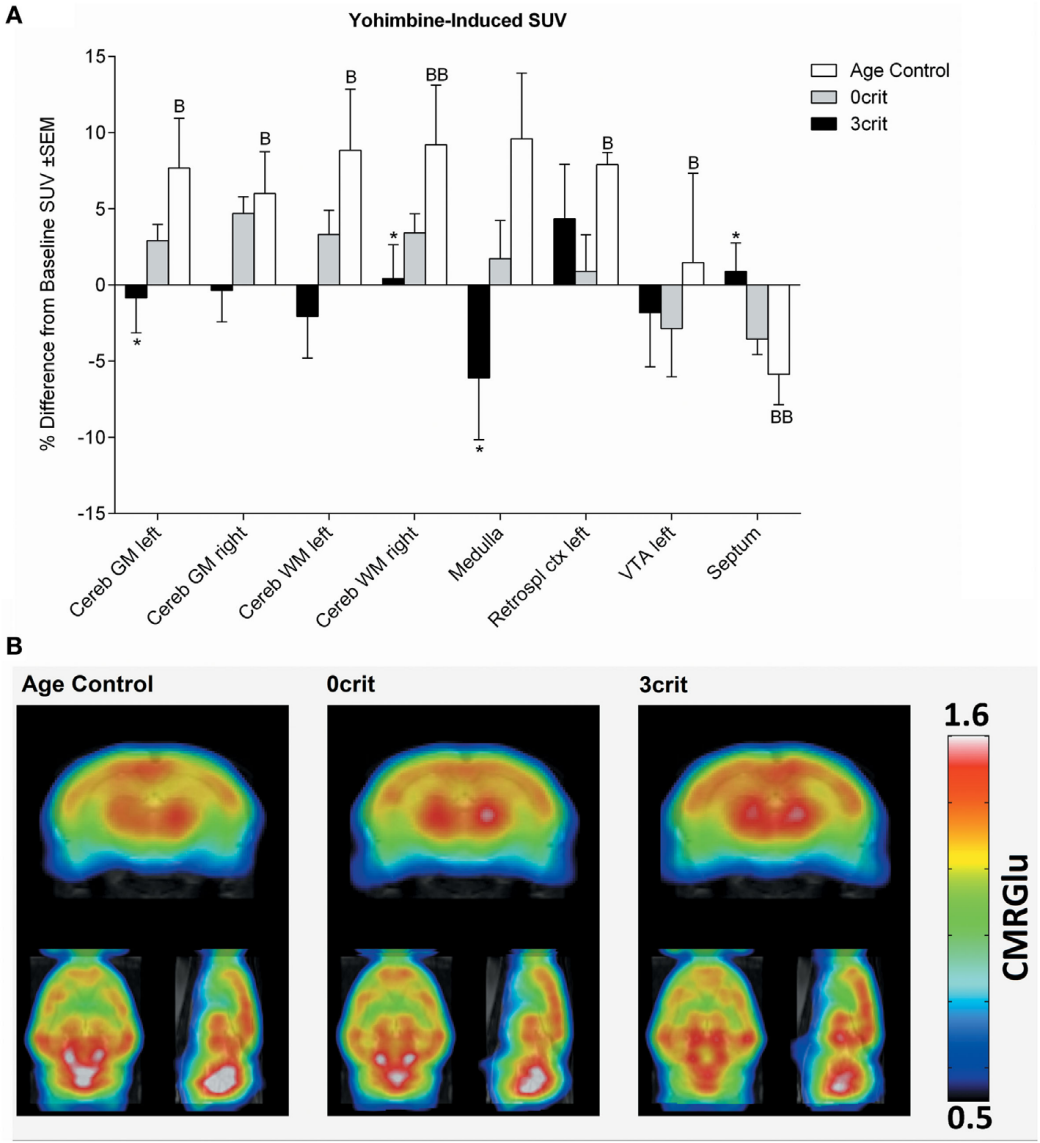
Regional quantification of metabolic activity after infusion of a yohimbine bolus (0.05 mg/kg i.v.). **(A)** Yohimbine-induced SUV expressed as relative difference from baseline (i.e., SUV measured after saline injection). Yohimbine increased SUV in several areas and decreased it in the septum of age-matched control rats not trained for cocaine self-administration. **(B)** Metabolic maps representing yohimbine-induced cerebral metabolic rate of glucose (CMRGlu) in Age Control, 0crit, and 3crit rats. *p < 0.05 vs Age Control, B *p* < 0.05, and BB *p* < 0.01 vs Baseline SUV. VTA, ventral tegmental area; Retrospl ctx, retrosplenial cortex; Cereb WM, white matter of cerebellum; Cereb GM, Gray matter of cerebellum.

Overall group and drug effects (either cocaine vs saline or yohimbine vs saline), and the group by drug interaction (*F* and *p*-values after Bonferroni correction) for each ROI is summarized in Table 1 and depicted in Figures 3–5.

**Table 1:** Summary of ANOVA of ROIs SUV.

	Cocaine						Yohimbine					
	*F* Drug	*p* Drug	*F* Group	*p* Group	*F* Dr × Gr	*p* Dr × Gr	*F* Drug	*p* Drug	*F* Group	*p* Group	*F* Dr × Gr	*p* Dr × Gr
Nucleus accumbens right	1.61	0.221	0.75	0.485	1.06	0.368	0.66	0.428	0.07	0.935	0.94	0.407
Nucleus accumbens left	1.73	0.205	0.85	0.444	1	0.387	1.43	0.247	0.42	0.662	0.04	0.958
Amygdala left	0.46	0.507	2.24	0.135	0.05	0.947	0.51	0.483	1.12	0.348	0.94	0.408
Amygdala right	0.61	0.446	2.08	0.153	0.66	0.528	0.97	0.337	3.12	0.069	0.95	0.407
Caudate and Putamen left	0	0.965	0.22	0.804	0.03	0.969	0	0.949	0.69	0.516	0.2	0.817
Caudate and Putamen right	0.02	0.876	**4.7**	**0.023**	0.9	0.423	2.31	0.146	2.89	0.082	2.35	0.124
Auditory cortex left	0.04	0.853	2.35	0.123	0.96	0.402	0.07	0.8	1.37	0.278	0.26	0.772
Auditory cortex right	1.77	0.2	0.21	0.814	0.83	0.453	2.21	0.155	0.02	0.976	0.65	0.532
Cingulate cortex left	**4.53**	**0.047**	1.46	0.258	1.34	0.286	2.38	0.141	0.5	0.618	1.44	0.264
Cingulate cortex right	2.59	0.125	0.83	0.453	0.26	0.776	1.58	0.226	1.27	0.304	0.57	0.573
Entorhinal cortex left	0	1	0.35	0.712	0.82	0.456	1.14	0.3	0.21	0.812	0.22	0.807
Entorhinal cortex right	1.39	0.254	0.6	0.559	0.87	0.437	0.92	0.349	3.03	0.073	0.83	0.451
Frontal association cortex left	0.1	0.753	**5.69**	**0.012**	0.36	0.705	0.59	0.454	1.13	0.345	2.3	0.129
Frontal association cortex right	0.13	0.725	0.87	0.436	0.16	0.857	0.19	0.668	0.25	0.778	1.46	0.259
Insular cortex left	1.2	0.288	1.19	0.327	0.58	0.572	1.95	0.18	1.91	0.177	0	0.998
Insular cortex right	0.23	0.636	1.42	0.266	0.5	0.617	0.43	0.523	0.68	0.52	1.46	0.259
Medial prefrontal cortex (mPFC) left	3.25	0.088	1.54	0.242	**3.88**	**0.04**	0.01	0.942	1.92	0.176	0.84	0.447
mPFC right	1.74	0.203	3.34	0.059	**3.72**	**0.044**	0.48	0.498	2.4	0.12	0.89	0.429
Motor cortex left	0.98	0.336	**6.01**	**0.01**	0.54	0.593	0.19	0.671	2.68	0.095	2.47	0.113
Motor cortex right	0.59	0.454	1.52	0.246	0.04	0.964	0.17	0.682	0.46	0.638	1.25	0.311
Orbitofrontal cortex left	0.13	0.725	2.91	0.08	0.15	0.865	1.11	0.306	1.46	0.259	1.09	0.359
Orbitofrontal cortex right	0.02	0.889	2.65	0.098	0.09	0.912	0	0.95	1.48	0.254	0.94	0.407
CortexParA left	0.44	0.517	2.09	0.152	1.64	0.222	**4.42**	**0.05**	0.47	0.635	1.03	0.378
CortexParA right	3.01	0.1	0.01	0.989	0.24	0.789	**6.76**	**0.018**	0.37	0.696	0.15	0.858
Retrosplenial cortex left	0.42	0.527	0.32	0.729	1.9	0.178	**7.03**	**0.016**	0.63	0.543	1.74	0.204
Retrosplenial cortex right	0.23	0.641	0.22	0.803	0.19	0.831	**5.15**	**0.036**	0.37	0.696	0.32	0.731
Somatosensory cortex left	0.04	0.843	**3.92**	**0.038**	0.48	0.628	0.11	0.739	2.95	0.078	1.86	0.184
Somatosensory cortex right	1.28	0.273	0.51	0.607	0.18	0.834	0.98	0.334	0.61	0.553	0.3	0.746
Visual cortex left	0.01	0.904	2.71	0.094	2.04	0.159	1.68	0.211	2.17	0.143	**3.76**	**0.043**
Visual cortex right	2.29	0.148	0.14	0.873	0.39	0.68	**4.61**	**0.046**	0.11	0.898	0.28	0.757
AnteroDorsal Hippocampus left	0.33	0.572	1.89	0.18	**4.94**	**0.019**	0.59	0.452	1.79	0.195	3.01	0.075
AnteroDorsal Hippocampus right	0.12	0.731	0.78	0.474	0.18	0.833	0.35	0.56	0.2	0.822	0.73	0.497
Posterior hippocampus left	0.07	0.799	2.61	0.101	0.13	0.875	0.02	0.901	1.96	0.169	0.23	0.794
Posterior hippocampus right	0.44	0.518	0.84	0.45	0.41	0.671	0.76	0.394	2.41	0.118	0.46	0.637
Hypothalamus left	5.28	0.034	2.13	0.147	**4.34**	**0.029**	1.24	0.281	0.31	0.734	0.28	0.759
Hypothalamus right	1.15	0.298	0.88	0.431	**4.48**	**0.026**	1.29	0.27	0.06	0.945	0.1	0.906
Olfactory bulb left	0.69	0.417	2.04	0.158	0.53	0.597	3.56	0.075	1.86	0.184	0.27	0.764
Olfactory bulb right	2.04	0.17	0.94	0.408	0.77	0.479	3.5	0.078	1.71	0.209	0.06	0.942
Colliculus superior left	1.96	0.179	0.93	0.414	0.53	0.596	0.25	0.626	**3.8**	**0.042**	0.36	0.706
Colliculus superior right	0.17	0.689	0.78	0.474	0.39	0.682	0.9	0.355	1.71	0.208	0.22	0.805
Midbrain left	0.04	0.843	2.01	0.163	0.4	0.673	2.28	0.148	1.67	0.216	0	0.996
Midbrain right	1	0.331	0.37	0.693	0.14	0.869	0	0.967	0.61	0.556	2.06	0.157
Ventral tegmental area (VTA) left	**4.47**	**0.049**	1.39	0.274	0.3	0.746	0.66	0.428	1.47	0.255	0.21	0.812
VTA right	**4.47**	**0.049**	1.79	0.195	0.19	0.83	0	0.982	2.59	0.103	0.28	0.757
Cerebellum gray matter left	0	0.946	0.4	0.677	0.01	0.986	**5.18**	**0.035**	2.05	0.158	3.51	0.052
Cerebellum gray matter right	0.34	0.568	0.66	0.529	0.15	0.858	**8.55**	**0.009**	1.13	0.344	3.05	0.072
Cerebellum white matter left	0.19	0.666	0.05	0.955	0.02	0.979	3.34	0.084	1.59	0.232	**3.72**	**0.045**
Cerebellum white matter right	0.09	0.769	0.63	0.545	0.11	0.896	**8.18**	**0.01**	1.45	0.26	**3.09**	**0.07**
Inferior colliculus left	1.8	0.196	0.78	0.471	1.22	0.32	2.69	0.118	1.09	0.357	0.54	0.59
Inferior colliculus right	0.07	0.787	0.87	0.435	1.07	0.364	2.9	0.106	1.66	0.218	0.6	0.559
Thalamus left	0.56	0.463	0.61	0.552	0.99	0.392	0.4	0.533	0.35	0.711	0.33	0.722
Thalamus right	0.01	0.91	1.05	0.37	0.23	0.795	0.56	0.463	0.72	0.501	2.44	0.116
Pituitary gland	1.25	0.278	1.94	0.173	0.34	0.714	0.71	0.41	5.12	0.017	0.62	0.549
Cerebellum blood flow	0.05	0.825	0.01	0.992	0.25	0.78	0.99	0.334	1.76	0.2	2.41	0.118
Periaqueductal gray	0	0.968	0.09	0.917	0.1	0.902	0.21	0.653	1.59	0.232	0.78	0.473
Pons	2.19	0.156	0.58	0.571	1.64	0.222	0.63	0.437	0.02	0.98	2.43	0.117
Septum	0.01	0.927	0.25	0.78	0.29	0.753	**9.12**	**0.007**	4.09	0.034	**4.18**	**0.032**
Medulla	1.86	0.19	2.41	0.118	0.05	0.954	0.18	0.672	0.47	0.634	**4.24**	**0.031**

*Significant effects are highlighted in bold*.

#### Baseline Differences in SUV

In both left and right mPFC and in the right caudate putamen (CPu), we found higher SUV in the 0crit as compared to AC. In 3crit rats, this higher metabolic activity in the mPFC and CPu was not seen. In the left somatosensory cortex, left visual cortex, left motor cortex, and left frontal association cortex, we found a lower baseline SUV in 3crit as compared to AC and 0crit rats (Figure 3).

#### Cocaine-Induced Changes in SUV

As shown in Figure 4, injection of a cocaine bolus corresponding to the self-administration dose (0.8 mg/kg) produced an increase in glucose uptake bilaterally in the mPFC of cocaine naïve rats. Conversely, in the same group, cocaine induced a decrease of glucose uptake in the hypothalamus; this effect differed significantly from 3crit animals. However, in both 0crit and 3crit animals, cocaine failed to induce a significant change in glucose uptake.

#### Yohimbine-Induced SUV

As shown in Figure 5, injection of a yohimbine bolus induced a significant increase of SUV in the cerebellum, medulla, left retrosplenial cortex, and left ventral tegmental area (VTA), and a decrease in the septum in AC rats. This effect differed significantly from 3crit animals in the septum, medulla and cerebellum. However, similar to cocaine, in both 0crit and 3crit animals, yohimbine did not significantly change glucose SUV respect to baseline (i.e., SUV measured after injection of a saline bolus).

### Correlation between Behavioral Addiction Sub-Dimensions and Regional Glucose Uptake

In a previous translational neuroimaging study, we demonstrated that the behavioral criteria characterizing 0crit and 3crit correlate with gray matter volume (GMV) within specific neuroanatomical clusters (23). Therefore, in both 0crit and 3crit conditions, we correlated metabolic activity with each behavioral criterion for each ROI. Interestingly, correlations surviving Bonferroni corrections were almost exclusively associated with the metabolic activity of 0crit rats. Specifically, under baseline conditions, the BP correlated positively with SUV in the VTA (left *r*^2^ = 0.7379, *p* = 0.006; right *r*^2^ = 0.7885, *p* = 0.003). Cocaine-induced SUV within the right frontal association (*r*^2^ = 0.8154, *p* = 0.002), right medial prefrontal (*r*^2^ = 0.8118, *p* = 0.002), and right orbitofrontal cortices (*r*^2^ = 0.7534, *p* = 0.005) correlated positively with persistence of cocaine-seeking. Yohimbine-induced SUV within the cingulate cortex (left *r*^2^ = 0.7569, *p* = 0.005; right *r*^2^ = 0.7413, *p* = 0.006) and anterodorsal hippocampus (*r*^2^ = 0.8686, *p* = 0.001) correlated negatively with resistance to punishment. In the 3crit condition, only one ROI survived Bonferroni correction—cocaine-induced SUV in the right VTA correlated negatively with resistance to punishment (*r*^2^ = 0.8968, *p* = 0.001).

### Enhanced Reinstatement Responses in Addicted Rats Are Negatively Correlated with Metabolic Activity in the VTA

In order to compare the effects of cocaine and yohimbine on brain metabolic activity with behavioral effects, reinstatement tests for cocaine-seeking behavior were performed. Two 3crit rats lost catheter patency during baseline training and therefore these tests were performed in eight 0crit and five 3crit animals.

**Figure 6:**
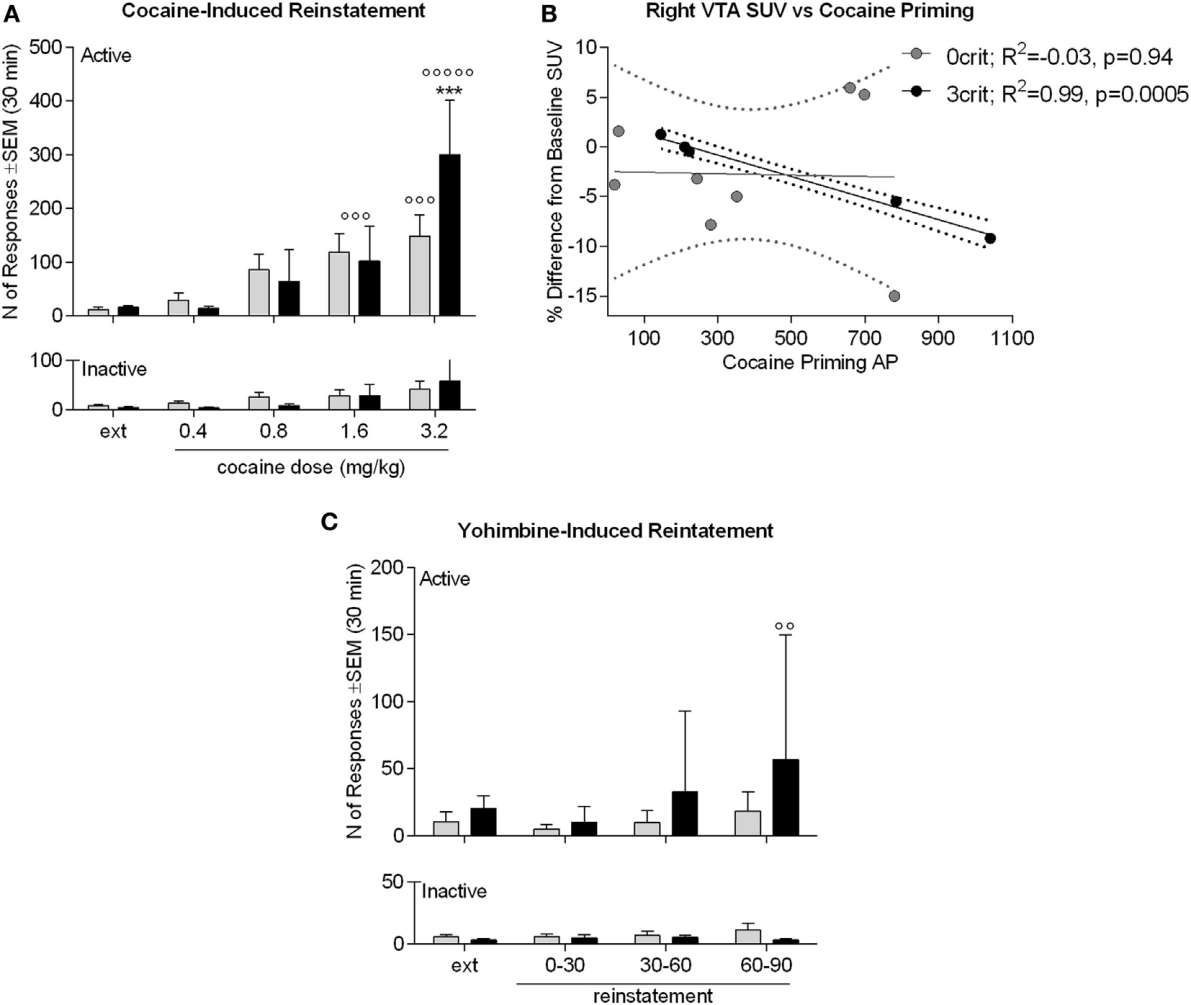
Cocaine and yohimbine-induced reinstatement in 0crit (*n* = 8) and 3crit (*n* = 5) rats. **(A)** Cocaine-induced reinstatement. Groups did not differ in number of active poking during the last 30 min of extinction (ext). The 120 min of the reinstatement phase are shown in 30 min time bins, each following the infusion of a dose of cocaine (0.4, 0.8, 1.6, 3.2 mg/kg). The dose of 1.6 mg/kg reinstated active poking in both 0crit and 3crit and the dose of 3.2 mg/kg further increased active poking selectively in 3crit. **(B)** 3crit, but not 0crit, show a negative correlation between total number of active poking produced during reinstatement and change in SUV relative to baseline produced in the right ventral tegmental area (VTA) by the i.v. injection of a cocaine bolus. **(C)** Yohimbine-induced reinstatement. Groups did not differ in number of active poking during the last 30 min of extinction (ext). Administration of a 0.5 mg/kg yohimbine dose failed to reinstate cocaine-seeking except for a trend shown by 3crit 60–90 min after yohimbine challenge. Data are shown as mean ± SEM; ***p < 0.001 vs 1.6 mg/kg dose; °°*p* < 0.01 and °°°*p* < 0.001 vs extinction.

An ANOVA of cocaine-induced reinstatement (Figure 6A) revealed a significant nose-pokes × dose × group interaction [*F*(4,44) = 2.61; *p* < 0.05]. Newman–Keuls revealed that 3crit and 0crit rats did not differ in active nose-pokes during extinction and that following the dose of 1.6 mg/kg, both 3crit and 0crit rats reinstated cocaine-seeking (*p* < 0.001). A dose of 3.2 mg/kg further increased cocaine-seeking in 3crit (*p* < 0.001 vs 1.6 mg/kg) but not 0crit rats. At this dose, 3crit rats had a higher reinstatement than 0crit rats that approached significance (*p* = 0.07). In 3crit but not 0crit rats, cumulative active poking during reinstatement was negatively correlated with cocaine-induced change in SUV in the right VTA (*r*^2^ = 0.9889, *p* = 0.0005) (Figure 6B).

### Yohimbine-Induced Reinstatement

An ANOVA of yohimbine-induced reinstatement (Figure 6C) revealed no nose-pokes × time bins x group interaction [*F*(3,33) = 1.18; *p* > 0.05]. However, there was an effect of time bins [*F*(3,33) = 3.11; *p* < 0.05]. Therefore, we run anyway a *post hoc* analysis that indicated a significant increase in active nose-poke compared to extinction (*p* < 0.01) during the third reinstatement time bin by 3crit rats. In addition, in contrast to 0crit rats, during the third reinstatement time bin, active pokes were significantly higher than inactive pokes in 3crit rats. Thus, data suggested a tendency of 3-crit animals to exhibit enhanced yohimbine-induced reinstatement responding. However, this result should be taken cautiously as only five 3crit rats were tested and yohimbine usually produces great variability (21), as was the case here.

## Discussion

Here, we subjected rats characterized for addict-like and non-addict-like behavior and age-matched cocaine-naïve control rats to FDG-PET acquisition to study alterations in glucose uptake associated with non-addicit- vs addict-like drug intake resulting from cocaine-taking behavior. We found a reduced glucose metabolic rate in 3crit rats relative to cocaine-naïve control animals in several cortical areas, including somatosensory, motor, visual and parietal association cortices. This result is in line with human data showing reduced frontal glucose metabolism in cocaine addicted patients (3, 4), supporting the construct validity of the 0/3crit animal model for cocaine addiction.

Region-specific alterations in metabolic brain activity have been used in numerous studies for the identification of biomarkers for disease states. Our goal was the identification of potential metabolic biomarkers indicative of addictive behavior. Here, our translational approach seems to be well suited as it allows the direct comparison of subjects that exhibit controlled drug intake vs. those that exhibit addict-like behavior under identical environmental conditions and levels of drug intake—variables that are impossible to control for in human studies. Therefore, our model allows for the disentanglement of biological features associated with addiction and resilience to addiction that are independent from the quantity of cocaine self-administered. We found higher CMRGlu in the mPFC of 0crit rats relative to cocaine-naïve control rats. This adaptation that was not present in 3crit animals. The mPFC is important for inhibitory control and action-outcome evaluation both in human and rodents, and mPFC impairment is a hallmark of addictive behavior (35, 36). Poor behavioral inhibition results from hypoactivity within the prefrontal cortex. mPFC hypoactivity is associated with a loss of synaptic plasticity in 3crit rats (29). In addition, optogenetic activation of the prelimbic (PL) cortex reversed compulsive CSA in addict-like rats while inhibition induced compulsive self-administration in non-addict-like rats (37). We propose that impaired inhibitory prefrontal control in cocaine addicts is also reflected by a loss of metabolic activity. In contrast, in individuals that exhibit prefrontal inhibitory control over drug intake, enhanced metabolic activity is observed. Therefore, the loss of metabolic prefrontal activity seems to be indicative of addictive behavior whereas the increase of prefrontal activity is associated with resilience to addiction, this suggests that development of therapies should aim in reverting prefrontal hypo-metabolism in addicts.

The rat mPFC is a heterogeneous structure that can be dichotomized in dorsal (centered in the PL region) and ventral [centered in the infralimbic (IL) region] parts. This anatomical dichotomization seems to be reflected by findings which show that the PL cortex plays an inhibitory control over drug self-administration whereas the IL seems to be recruited more in extinction phases, although this dichotomization could be too simplistic [for a comprehensive review, see Ref. (38)].

In clinical practice, neuroimaging biomarkers that indicate addictive behavior are costly and blood biomarkers provide an alternative. Indeed cocaine addicts showed altered blood level of cytokines and brain-derived neurotrophic factor (39–41) and those peripheral biomarkers may also correlate with cognitive performance (42). For instance, the plasma level of IL-6 was associated with poor executive function in cocaine addicts (43). This suggest that in future clinical practice, to achieve a comprehensive characterization of the patient’s addictive state, the search of neuroimaging biomarkers of addiction could be integrated or preceded by less expensive blood and cognitive analyses, a line of research worth being pursued.

We also studied the effects of a cocaine challenge on glucose uptake in our three groups of rats. Cocaine increased glucose uptake in the mPFC in cocaine-naïve control rats but had no such effect in 3crit and 0crit rats. In rats, it has been shown that cocaine-induced increases in brain metabolic activity can undergo tolerance (44). The development of tolerance to the effects of cocaine is a determinant of the maintenance of CSA that can develop under both contingent and non-contingent regiments of administration (45). This suggests that the lack of increase in glucose uptake induced by cocaine in the mPFC observed in both 0crit and 3crit rats is a phenomenon associated with repeated exposure to cocaine, but not necessarily indicative of cocaine addiction.

Cocaine produces its effect mainly by indirect potentiation of dopamine transmission; however, cocaine is a non-selective monoamine transported blocker (17) and other monoamine systems such as norepinephrine may contribute to cocaine’s effect on glucose metabolism. Therefore, we tested the effect of yohimbine on brain glucose utilization in our rats. Yohimbine is a α2 adrenergic receptor antagonist often used as pharmacological stressor in addiction research, as it indirectly elevates norepinephrine levels (18, 20). Our data indicate that in cocaine-naïve control rats, yohimbine increases glucose SUV in several areas, with the exception of the septum, in which glucose uptake was decreased. Conversely, in both 0crit and 3crit rats, yohimbine did not significantly alter brain glucose uptake. This effect is similar to what we have observed following a cocaine challenge and seems to be related to the intake of cocaine rather than addictive behavior. One could thus speculate that repeated cocaine exposure may have induced tolerance to the effect of elevated extracellular norepinephrine on brain glucose uptake. This, together with similar results obtained by cocaine challenge, suggests that cocaine affects brain glucose uptake not only through dopamine but also with the contribution of other monoamines such as norepinephrine.

Cocaine-induced reinstatement of cocaine-seeking in 3crit rats was more pronounced than in 0crit rats; this enhanced responding correlated negatively with cocaine-induced metabolic activity in the VTA. Interestingly, we also found in 3crit animals that VTA glucose uptake following a cocaine challenge was negatively correlated with resistance to punishment (*r*^2^ = 0.89, *p* = 0.001). Together these results suggest that a decrease in glucose uptake in the VTA induced by cocaine is part of the mechanism causing compulsive cocaine-seeking behavior. Indeed, it has been shown that low glucose levels can increase firing of dopamine neurons in the VTA (46), a neurochemical event that can promote drug-seeking behavior in 3crit animals (47, 48).

In a previous translational neuroimaging study, we demonstrated that within specific neuroanatomical clusters, the three behavioral sub-dimensions (persistence of drug-seeking, motivation for cocaine, and resistance to punishment) of addiction show divergent correlations with GMV in 0crit relative to 3crit rats (23). Here, we ran correlational analyses between SUV and the three addiction criteria to determine whether we could replicate this result with glucose uptake. We found several correlations that were less extensive and located in different ROIs with respect to those we found with GMV (23). This suggests that there is not a direct relationship between GMV and glucose metabolism, at least with respect to their contribution to the behavioral sub-dimensions of cocaine addiction scored by the 0/3crit model. Interestingly however, we found that the correlations observed were present only in 0crit rats, which is in line with our structural neuroimaging data (23). Indeed, even in the absence of absolute differences, the ratio between a functional, or structural, variable and a behavioral score determines the difference in cocaine-seeking between resilient and addict-like rats.

This study leaves unanswered the question on whether the differences observed between 0crit, 3crit and cocaine naïve rats developed over cocaine history or pre-existed. This question will be addressed in a future longitudinal study. However, the choice of a cross-sectional approach, like similar preclinical FDG studies before [(12); see also comparison of short and long cocaine access in Ref. (13)], did not hamper the contribution of the present study to the progress of knowledge as we could establish experimental conditions that are impossible to obtain in humans. Indeed, our cocaine-naïve controls were exposed to identical life conditions of cocaine-exposed rats, and the non-addict-like (0crit) rats consumed an amount of cocaine comparable to addict-like rats (0crit). Both these conditions are impossible to obtain in clinical studies and allowed us to get rid of biases derived from different life conditions and total life-span cocaine consumption, which represents the added value of this study independently on whether group differences pre-existed or developed over time.

In conclusion, rats maintaining control over drug intake are characterized by higher glucose utilization in the mPFC and CPu respect to cocaine-naïve rats—this adaptation in metabolic activity is not present in addict-like rats. These findings reveal potential biomarkers for controlled and addictive cocaine intake. Metabolic biomarkers would be of great help for clinical treatment development (49). Thus an intervention, whether pharmacological, behavioral, or neuromodulatory, should be able to restore metabolic prefrontal activity, which would then be indicative of regaining control of cocaine-taking behavior. Future clinical studies should explore whether similar metabolic alterations are observed in recreational vs. addicted cocaine users.

## Ethics Statement

Experimental procedures were in accordance with the NIH ethical guidelines for the care and use of laboratory animals and conform to the EU Directive 2010/63/EU for animal experiments, and were approved by the local animal care committee (Regierungspräsidium Karlsruhe, Germany).

## Author Contributions

NC, AC-L, and RS were responsible for the study design and wrote the manuscript. MR contributed to manuscript writing. NC coordinated the study. AC-L analyzed PET data. NC and TT conducted behavioral tests, assisted with PET acquisition, collected and analyzed behavioral data. MR and NV conducted, and BW supervised, PET acquisition and images reconstruction.

## Conflict of Interest Statement

The authors declare that the research was conducted in the absence of any commercial or financial relationships that could be construed as a potential conflict of interest.
